# Photopolymer-Based Composite with Substance Release Capability Manufactured Additively with DLP Method

**DOI:** 10.3390/ma17020322

**Published:** 2024-01-09

**Authors:** Dorota Tomczak, Sławomir Borysiak, Wiesław Kuczko, Ariadna B. Nowicka, Tomasz Osmałek, Beata Strzemiecka, Radosław Wichniarek

**Affiliations:** 1 Institute of Chemical Technology and Engineering, Poznan University of Technology, Berdychowo 4, 60-965 Poznan, Polandslawomir.borysiak@put.poznan.pl (S.B.); beata.strzemiecka@put.poznan.pl (B.S.); 2 Faculty of Mechanical Engineering, Poznan University of Technology, Piotrowo 3, 61-138 Poznan, Poland; wieslaw.kuczko@put.poznan.pl; 3 Faculty of Materials Engineering and Technical Physics, Poznan University of Technology, Piotrowo 3, 60-965 Poznan, Poland; 4 Department of Pharmaceutical Technology, Poznan University of Medical Sciences, 3 Rokietnicka St., 60-806 Poznan, Poland; tosmalek@ump.edu.pl

**Keywords:** digital light processing, composite, photopolymer, resin, caffeine, release

## Abstract

In this study, caffeine-loaded photoresin composites with homogeneous structures, suitable for additive manufacturing of transdermal microneedle systems, were obtained. The properties of the composites with varying caffeine concentrations (0.1–0.4% *w*/*w*) were investigated for carbon–carbon double bond conversion using Fourier Transform Infrared Spectroscopy, surface wettability and mechanical properties using a static tensile test and nanoindentation, and caffeine release in ethanol using UV-Vis. The caffeine concentration did not affect the final degree of double bond conversion, which was confirmed in tensile tests, where the strength and Young’s modulus of caffeine-loaded samples had comparable values to control ones. Samples with 0.1 and 0.2% caffeine content showed an increase in nanohardness and reduced elastic modulus of 50 MPa and 1.5 MPa, respectively. The good wettability of the samples with water and the increase in surface energy is a favorable aspect for the dedicated application of the obtained composite materials. The amount of caffeine released into the ethanol solution at 1, 3 and 7 days reached a maximum value of 81%, was higher for the lower concentration of caffeine in the sample and increased over time. The conducted research may enhance the potential application of composite materials obtained through the digital light processing method in additive manufacturing.

## 1. Introduction

Nowadays, the personalization of drug dosage forms and medical devices is one of the most important developments in the medical and pharmaceutical industry. The individual needs of patients can be met by manufacturing products tailored to their bodies in terms of biocompatibility, geometry, drug content, biochemical profile, type and localization of the disease, etc. One of the methods enabling the manufacturing of such products is 3D printing based on digital light processing (DLP). During the process, a liquid photopolymer resin is cross-linked layer by layer according to the digital design of the object being produced. The DLP method allows for the processing of various photopolymer materials and their mixtures with other substances, resulting in composite structures where the photopolymer resin serves as the matrix. The selection of processing parameters is crucial and includes factors such as the exposure time for each layer, layer thickness, printbed (area) offset, and number of transition layers [1]. Compared to other additive manufacturing methods, the DLP method offers several advantages, including the ability to produce complex objects with smaller dimensions and high production repeatability. In addition, it is possible to use a smaller layer thickness compared to most additive manufacturing methods, which has a beneficial effect on the interactions between layers and ultimately on the performance of the products [2]. Thanks to its advantages, the DLP method has found applications in many fields of technology, including medical and pharmaceutical applications [3,4].

The literature provides examples of the use of the DLP in the manufacture of various drug dosage forms, including tablets with high drug loading and sustained release. The DLP method was used in the production of methacrylate and polyethylene glycol-based tablets with variable thickness and variable loading of atomoxetine to modify the release from immediate to prolonged [5]. Similar studies have been conducted for ibuprofen by changing the content of excipients and printing parameters [6]. DLP does not require heating, making it highly applicable for thermosensitive but UV-resistant APIs (Active Pharmaceutical Ingredients) [7]. It is also possible to produce bilayer tablets, as confirmed by research performed on printed tablets based on hydrochlorothiazide and warfarin sodium [8]. The work of Mau, R. et al. [9] evaluated the mechanical properties in compression and the drug release ability of systems consisting of photopolymer and dexamethasone for use as implants. In addition, DLP printing is also being used in bioprinting, where it has applications in tissue repair and regeneration, disease modeling or drug screening [10].

Microneedle array can be considered a special type of transdermal drug delivery system that also can be obtained using the DLP method. Microneedle arrays deliver the drug by creating microchannels in the *stratum corneum*, allowing the drug to overcome the hydrolipid barrier and increase its bioavailability. The height of the microneedles should be long enough to reach the layer of skin called the epidermis but short enough not to irritate the patient’s pain receptors [11]. Both the parameters of the process and the chemical composition of the resins used to manufacture microneedles affect their final geometry [12]. There are five basic types of microneedles: solid, hollow, coated, dissolvable and hydrogel-type [13]. The DLP method is known to be used to obtain microneedle systems using, for example, polyethylene glycol diacrylate (PEGDA) as the matrix and the drug diclofenac sodium [14] or gelatin methacryloyl and the drug amoxicillin [15], where PEGDA and gelatin methacryloyl swell, enabling drug release by diffusion. Moreover, there are known microneedle systems manufactured using UV light in different methods than DLP printing, like casting PEGDA into a silicone vessel and exposing to UV light [16] or using Projection Micro-Stereo Lithography, where microneedles were additionally coated with the anticancer drug gemcitabine and sodium carboxymethyl cellulose [17] with the potential for topical drug delivery in personalized therapies.

The aim of this work was to obtain samples of acrylic-urethane resin loaded with caffeine, which, when cross-linked to a certain degree, swell in the presence of a liquid medium, so it can act similarly to hydrogels, releasing the active substance through diffusion. Caffeine, as a representative of a primarily hydrophilic therapeutics, is used as a dietary supplement [18] or a therapeutic agent showing analgesic, antioxidant, neuroprotective effects [19]. This substance can be obtained from coffee beans, tea leaves or kola nuts [20]. To date, no resin composites with caffeine, or any other substance with a polarity significantly different from that of the matrix, have been obtained with a homogeneous structure and potential for use as a microneedle transdermal system. The composite material’s suitability for use in transdermal drug delivery systems was assessed through tests of its tensile strength, nanoindentation, degree of cross-linking, and degree of drug release. Additionally, the impact of the active ingredient on the composite’s final properties at both the macro and nano scale, was evaluated.

## 2. Materials and Methods

### 2.1. Materials

An acrylate-urethane resin (Phrozen Technology, Hsinchu, Taiwan) consisting of acrylate oligomer, acryloyl morpholine, light stabilizer bis(1,2,2,6,6-pentamethyl-4-piperidyl) sebacate and photoinitiator diphenyl(2,4,6-trimethyl benzoyl) phosphine oxide was used to prepare the samples. The resin properties relevant to the production of composite samples with caffeine using the DLP method are solubility in ethanol, insolubility in water, density of 1.11 g/cm^3^, boiling point of 200 °C and viscosity of 50–100 cps [21].

The caffeine used for the composite samples had a purity of 99% (Pol-Aura, Warsaw, Poland). The solubility of caffeine in ethanol was determined as 0.02 g/mL at 75 °C [22].

The ethanol used to dissolve caffeine had a concentration of 96% (Avantor Performance Materials Poland, Gliwice, Poland).

### 2.2. Production of Composites

To produce the composites, three caffeine solutions were obtained: 50, 100 and 200 mg of caffeine were poured into 10 mL of ethanol, which were annealed at 75 °C for 2 h until the caffeine was completely dissolved. The obtained solutions were then added to 50 g of liquid resin, obtaining solutions with caffeine concentrations of 0.1%, 0.2% and 0.4%, which were designated AKA05, AKA1 and AKA2. The obtained resin-based solutions were stirred using a magnetic stirrer (Heidolph, Schwabach, Germany) with a rotational speed of 500 rpm and a table temperature of 50 °C preventing caffeine from crystallizing in the solution. In addition, a control solution was prepared with 50 g of resin and 10 mL of ethanol, designated AKA0, and 50 g of pure resin, designated A0. The designations of the samples and the composition of the solutions for sample preparation are given in Table 1.

Samples were printed from solutions of resin, ethanol and caffeine, as well as pure resin and resin with ethanol, using a Phrozen Sonic Mini 8k DLP printer (Phrozen Technology, Taiwan) with the following parameters: layer height 0.05 mm; bottom exposure time 35 s; exposure time 10.5 s; transition layer count 6; transition type linear; bottom layer compensation a = 0 mm, b = −0.04 mm. The samples were rectangular in shape with dimensions of 2 × 10 × 80 mm. The parameters were saved and stored in files prepared using Chitubox software, version 1.9.3 (CBD Technology Co., Ltd., Shenzhen, China). A total of 25 samples were acquired for each series of samples A0, AKA0, AKA05, AKA1, and AKA2.

After printing, the samples were cleaned in a Creality UW-01 washing machine (Shenzhen Creality 3D Technology Co., Ltd., Shenzhen, China) by washing in ethanol for 2 min. The samples were then post-processed through UV irradiation in a UV Curing Chamber for 10 min (XYZPrinting, New Taipei, Taiwan).

### 2.3. FTIR-ATR Measurement

The Vertex 70 spectrometer (Bruker, Karlsruhe, Germany) was used during Fourier Transform Infrared Spectroscopy (FTIR) measurements with the following parameters set: 64 scans with a resolution of 4 cm^−1^ in the range of 4000–400 cm^−1^. The studied materials were measured using the ATR technique, with diamond crystal, produced by Pike Technologies, USA. The percentage of acrylic double bond (C=C) conversion (DC) was calculated using Equation (1):

(1) $$DC\left(\%\right)=\frac{{\left(A_{1407}/A_{1722}\right)}_{0}-{\left(A_{1407}/A_{1722}\right)}_{t}}{{\left(A_{1407}/A_{1722}\right)}_{0}},$$

where ${\left(A_{1407}/A_{1722}\right)}_{0}$ and ${\left(A_{1407}/A_{1722}\right)}_{t}$ are relative absorbance (peak area ratio) of C=C before and after crosslinking, respectively.

### 2.4. Wettability Measurement

The wettability of the samples was determined through contact angle values using two substances: water and polyethylene glycol 200 (Sigma-Aldrich, Saint Louis, MO, USA) using the hanging drop method with the aid of an OCA 20 Optical Goniometer apparatus (Dataphysics, Filderstadt, Germany). The surface energy (SE) consisting of two components, polar and dispersion, was calculated using the Owens–Wendt method presented by Equation (2):

(2) $$\gamma_{SL}=\gamma_{S}+\gamma_{L}-2\left(\sqrt{\gamma_{S}^{d}\gamma_{L}^{d}}-\sqrt{\gamma_{S}^{p}\gamma_{L}^{p}}\right),$$

where $\gamma_{SL}$—SE on the solid–liquid interface; $\gamma_{S}$—SE of solid; $\gamma_{L}$—SE of liquid; *d*—dispersive component; and *p*—polar component.

### 2.5. Evaluation of Caffeine Release by UV-Vis

The degree of caffeine release from the samples was evaluated by soaking 0.3 g (approximately 10 × 10 × 2 mm) cut pieces of the samples in 10 mL of ethanol placed in a polypropylene test tube with a plug for 1, 3 and 7 days. An obtained solution was then tested using UV-Vis to determine the caffeine content. A V-630 UV-Vis spectrometer made by JASCO, Japan, was used. The measurements were performed in the range of 220–350 nm.

### 2.6. Tensile Testing

The tensile test was performed in accordance with the ISO 527 standard [23] for all series of specimens on a Zwick/Roell Z020 testing machine (Zwick Roell, Ulm, Germany). The load capacity was 20 kN and the tensile rate was 1 mm/min. The following parameters were obtained: tensile strength (σ_m_), relative elongation (ε_B_) and Young’s modulus (E).

### 2.7. Nanoindentation

The nanoindentation test was carried out using Nanomechanics iMicro apparatus (KLA Company, Milpitas, CA, USA) with a load equal to 50 mN. The nanohardness parameter (nH) was determined, and the reduced elastic modulus (*E_R_*) was calculated using Formula (3):

(3) $$E_{R}=\frac{\sqrt{\pi }}{2}*\frac{S}{\sqrt{A}}$$

where *A*—contact area, and *S*—measured stiffness.

## 3. Results

### 3.1. Evaluation of the Structure and Composition of the Samples

**Figure 1:**
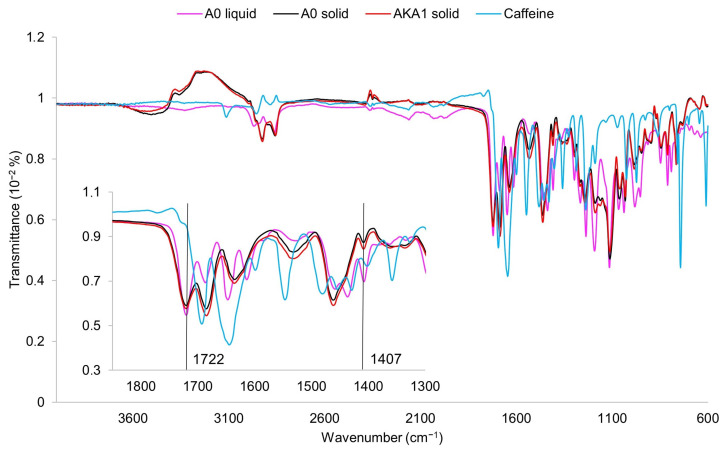
FTIR-ATR spectra for resin before crosslinking (A0 liquid), resin after crosslinking (A0 solid), resin with 100 mg caffeine and ethanol (AKA1 solid), and caffeine (caffeine).

Figure 1 shows the spectra corresponding to the uncrosslinked and crosslinked resin. For the acrylic-urethane resin, the main bonds involved in crosslinking are the double bonds between the carbons (C=C) in the acrylate groups. These correspond to the peaks visible on the A0 liquid spectrum for wavenumbers 1647, 1407, and 808 cm^−1^ [24,25,26]. When comparing the spectra for crosslinked samples of pure resin and resin with caffeine, no new peaks or significant change in intensity can be seen, which positively demonstrates the negligible effect of caffeine on the crosslinking process.

The intensity of the peaks corresponding to the C=C acrylic bonds decreases after the printing process, which is present for the A0 solid and AKA1 solid samples with respect to the A0 liquid sample, indicating the disappearance of double bonds and crosslinking of the structure. To determine the degree of double bond conversion based on the change in peak area, data for peaks with a wavenumber of 1407 cm^−1^ were used, since only this peak among the three corresponding to C=C bonds after the crosslinking process does not overlap with any other peak from other bonds. In addition, the reference peak for wavenumber 1722 cm^−1^ corresponding to the carbon-oxygen C=O double bond [25], which does not change during the crosslinking process, was used in the calculations.

**Figure 2:**
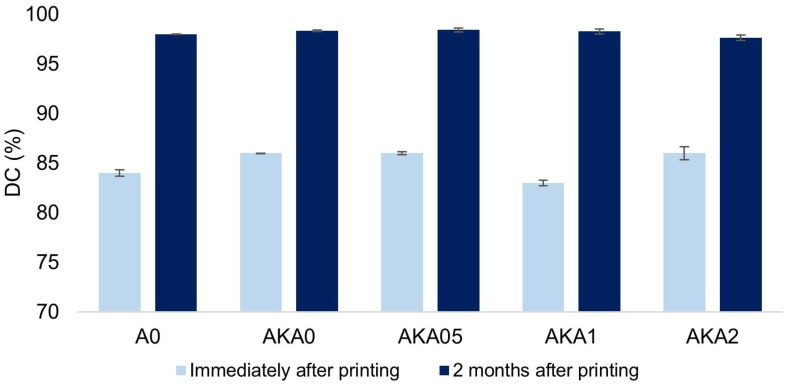
The dependence of the degree of cross-linking (DC) of resin samples on caffeine and ethanol content and time of measurement.

Figure 2 shows the dependence of double bond conversion on the caffeine content of the sample. Measurements were made immediately after printing the samples, as well as 2 months later. The percentages of bond conversion for samples just after printing and postprocessing are comparable and are in the range of 84–86%. Thus, it can be seen that caffeine does not significantly affect the degree of crosslinking of the resin. Also, no correlation can be seen between increasing the concentration of caffeine in the sample and the degree of crosslinking, which is a positive aspect in terms of preserving the original properties of the resin. Similarly, in the case of double bond conversion measurements after two months, the values of the degree of bond conversion increased to about 98% for each sample. This proves the inadequacy of the applied postprocessing in the form of UV irradiation with the device used to achieve the maximum degree of resin cross-linking in a shorter time. 

**Figure 3:**
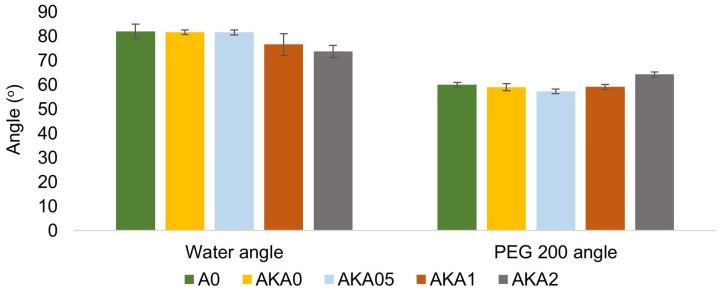
Wetting angle values using water and PEG 200 for resin, resin with ethanol and resin–ethanol–caffeine samples.

**Figure 4:**
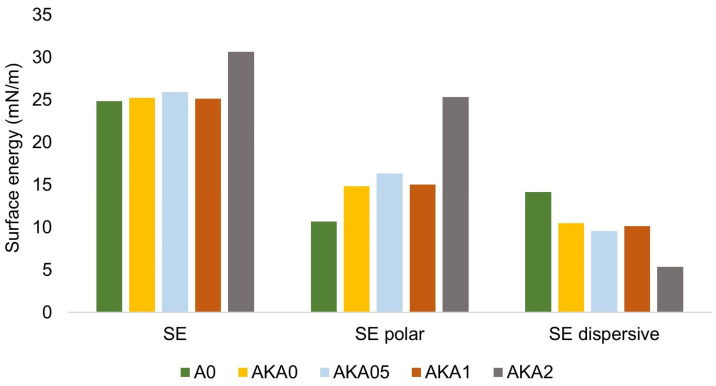
Surface energy values along with polar and dispersion component values for resin, resin with ethanol and resin–ethanol–caffeine samples.

Figure 3 and Figure 4 show the results of wettability measurements using the hanging drop method. The values of the wetting angle of the drop on the surface of the samples using water slightly decrease with increasing caffeine content. For PEG 200 liquid, the amplitude between the results for samples without and with caffeine is 5°, so it can be said that the results are comparable. However, it can be seen that there is an increase in the surface energy for sample AKA2 relative to the other samples, as well as an increase in the polar component and a decrease in the dispersive component of the energy with increasing caffeine content in the samples. The described observations are caused by the predominantly hydrophilic nature of caffeine, whose presence in the composite samples improves their water wettability. In terms of drug release from the resin in contact with the human body, this is a favorable aspect that may improve caffeine migration in the presence of water from human skin.

### 3.2. The Drug Release Process

**Figure 5:**
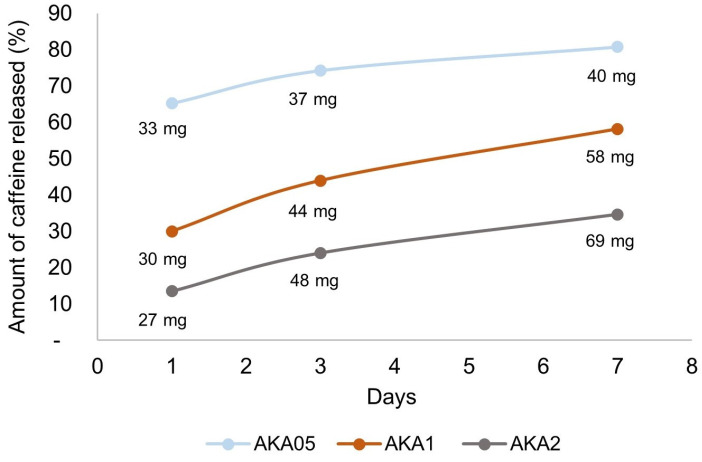
Caffeine release from samples over time profiles.

The profile of caffeine release from the samples at 1, 3 and 7 days is shown in Figure 5. The error values for the results obtained were less than or equal to 3%, so they were not presented graphically to increase the readability of the graph. After the first day, the percentage amount of released caffeine was 14, 30 and 65% for samples AKA2, AKA1 and AKA05, respectively, which was calculated by relating the values of 27, 30 and 33 mg of released caffeine to the original caffeine content of the sample. The amount of caffeine released after 1 day for each sample is similar at around 30 mg. After 3 and 7 days, it can be seen that the more caffeine there is in the sample, the more that is released. After 7 days, the amount of caffeine released increases by an average of 20 percentage points for each sample relative to the first day of release, reaching a maximum value of 81% for sample AKA05. The difficulty in achieving 100% caffeine release is probably caused by the limited solubility of caffeine in ethanol and the establishment of an equilibrium between the caffeine concentration in the sample and the concentration in ethanol. When samples are soaked, ethanol penetrates deep into the sample’s structure, allowing this equilibrium to change in favor of a higher caffeine concentration in the ethanol surrounding the sample, thus allowing more caffeine to be released from the sample through diffusion along with an increase in the time the sample is held in ethanol, which is nevertheless not enough to release all of the caffeine from the samples. To be sure of the cause of the limited caffeine release from the samples, additional studies should be conducted. This is an aspect that opens up research threads related to improving the release rate from photoresin-based composites loaded with non-crosslinking substances.

### 3.3. Mechanical Properties of Composites

The values of Young’s modulus (E), tensile strength (Rm) and relative elongation (A) for the samples tested immediately after printing and 2 months later are shown in Table 2 and Table 3. It can be noted that the addition of ethanol causes an approximately twofold decrease in the mentioned parameters compared to the properties of the pure resin. This is probably due to the effect of ethanol on the termination of the radical polymerization process of the resin through the chain transfer reaction to the solvent, which could result in the acceleration of the reaction kinetics depending on the activity of the solvent-derived radical, which would ultimately affect the shortening of the overall crosslinking process of the structure [27,28]. The exposure time of the samples, which was suitable for pure resin samples, may turn out to be too long for resin samples with added solvent by shortening the crosslinking reaction time, which would result in photodegradation due to the excessive radiation intensity adopted by the material at a certain time, justifying the deterioration of the strength properties. In addition, by introducing an additional substance such as ethanol, the interactions between fragments of the polymer chains were changed, which could also affect the mechanical properties. The mentioned factors are mainly responsible for the change in mechanical properties of the obtained resin–ethanol samples, but in order to determine which phenomena prevailed, additional studies related to the course of the radical polymerization reaction should be carried out. However, in measurements taken immediately after manufacturing of the samples, the effect of caffeine on the strength properties is not visible. 

After two months, there is a noticeable reduction in Young’s modulus of about 36 MPa and tensile strength of about 4 MPa for pure resin with unchanged elongation. In addition, there is a remarkable increase in Young’s modulus by 74 MPa for samples with ethanol, and about 20 MPa for samples with caffeine with almost constant tensile strength and elongation. Taking into account the perceived Young’s modulus measurement error values of 40–50 MPa for pure resin and 10–20 MPa for samples with ethanol and caffeine right after printing and 2 months later, a general conclusion can be drawn about the insignificant effect of the change in the degree of double bond conversion from 85 to 98% on the tensile properties of resin samples with ethanol or caffeine added, and the slight increase in stiffness of pure resin samples resulting from the full completion of the polymerization process within 2 months.

The reduced elastic modulus and nanohardness values determined by using a nanoindenter for samples tested two months after printing are presented in Table 4. The addition of ethanol results in a slight reduction in modulus, similarly to the macro-scale tests. The addition of caffeine at concentrations of 0.1 and 0.2% induces an increase in modulus of about 1.5 GPa, while increasing the concentration to 0.4% causes a decrease in modulus to a value comparable to the resin with ethanol control sample. A similar trend can be seen in the case of nano-hardness, which decreases by the addition of ethanol by 50 MPa, then by the addition of caffeine at concentrations of 0.1 and 0.2%, increases to a value comparable to the nano-hardness of pure resin, and decreases again when the concentration is increased to 0.4%. Thus, caffeine at a concentration of 0.1–0.2% to some extent has a strengthening effect by changing the mechanical parameters at the nanoscale, which may be caused by interactions between caffeine particles and resin particles, which are perhaps relevant only in a limited concentration range. This phenomenon is most beneficial in the context of the application of the developed material within transdermal microneedle systems. However, it is necessary to perform additional tests to confirm the described assumption.

### 3.4. Advantages and Potential of the Developed System

The presented research focuses on the developed resin–caffeine system, which has very high potential within the application for the production of transdermal microneedle systems. The proposed material, due to its ability to release the active substance, is similar to the hydrogel materials used for transdermal microneedle systems. Thanks to the studied release characteristics of caffeine, it can be seen that the amount of released substance is increasing with time. In terms of the use of microneedle systems with prolonged release for certain disease entities, this is an important advantage. The developed material is distinguished from other microneedle systems, such as the coated microneedle system, for which drug delivery is rapid and occurs with the dissolution of the drug coating in contact with the skin [29]. In addition, systems developed on the basis of resin–caffeine material probably could have an attached reservoir with the active ingredient prolonging its operation and increasing the amount of deliverable substance similarly to hydrogel systems, while avoiding blockage of drug delivery from the reservoir by clogging the delivery channel, as may be the case with hollow microneedles [30]. It is also possible to use active ingredients other than caffeine. The main limitation is ensuring that the cosolvent used in manufacturing of resin–drug composite is a solvent for both the selected resin and the active substance, which was fulfilled by the ethanol used in the selected work. As can be seen, the research conducted opens up many research paths concerning, among other things, increasing the amount of loaded drug in the resin, using different active substances, cosolvents and resins, or optimizing the micro-needle manufacturing process to obtain the desired strength and functional properties, including full release of substances from the system.

## 4. Conclusions

The results presented confirm the feasibility of successfully obtaining homogeneous composites from photopolymer resin and caffeine using the digital light processing method. A number of studies indicate that caffeine used in the concentration range of 0.1–0.4% has little effect on the key functional and structural properties of the composites. The proposed geometry of the specimens allowed the selected tests to be carried out according to the standards, leaving space for complementary tests on specimens containing microneedles.

The evaluation of carbon–carbon double bond conversion allows us to draw a conclusion about the lack of influence of caffeine or ethanol in the amounts used on the degree of double bond conversion of the samples. This is a favorable aspect in terms of preserving the inertness of caffeine during the resin crosslinking process, increasing the likelihood of maintaining the stability of its chemical structure and properties for pharmaceutical applications.

The tests also made it possible to evaluate the degree of caffeine release from samples immersed in ethanol at 1, 3 and 7 days. The maximum release rate of 81% was obtained for samples with the lowest caffeine concentration—0.1%—after a time of 7 days. Samples with higher caffeine concentrations achieved a lower release rate, most likely due to the limited solubility of caffeine in ethanol and the settling equilibrium of caffeine concentrations in the sample and surrounding ethanol. The results obtained indicate potential sites for expanding the study using a different matrix or release medium or taking into account other factors that modify the release of active substances from photo-curable resins.

The mechanical properties of the samples evaluated immediately after the printing process vary depending on the use of ethanol—samples made of pure resin have the highest strength parameters, while the addition of ethanol caused an approximate twofold reduction in Young’s modulus and strength values. After a period of 2 months, the strength properties practically did not change. It should be noted that the amount of caffeine used did not noticeably affect mechanical properties on the macro scale, but the strengthening effect of caffeine particles on the nanoscale can be observed by increasing the nanohardness for caffeine concentrations of 0.1 and 0.2%. Thus, an area of optimization of the process of obtaining samples limiting the influence of the solvent present in the sample on the deterioration of the mechanical properties of the composites emerges.

The results presented contribute to the state of the art of photopolymer-based composites as well as their functional and performance properties. In addition to the successes achieved in obtaining composite samples, including the preservation or improvement of the properties studied, places for the optimization and development of the research threads undertaken by the authors have also been identified, which is important in the context of future research conducted in the area of intensively developed DLP 3D printing.

## Figures and Tables

**Table 1:** Sample designations; caffeine and ethanol content in resin.

	Amount of Resin (g)	Amount of Ethanol (mL)	Amount of Caffeine (mg)
A0	50	-	-
AKA0	50	10	-
AKA05	50	10	50
AKA1	50	10	100
AKA2	50	10	200

**Table 2:** Parameters obtained by tensile testing of samples immediately after printing.

	E (MPa)	Rm (MPa)	A (%)
A0 AKA0 AKA05	778.0 ± 59	20.4 ± 0.3	18.9 ± 0.7
	361.0 ± 7.8	8.5 ± 0.2	11.2 ± 1.9
	343.5 ± 5.9	8.2 ± 0.3	9.2 ± 1.8
AKA1 AKA2	346.3 ± 7.4	8.3 ± 0.5	9.2 ± 2.8
	356.5 ± 12	8.6 ± 0.2	9.2 ± 2.3

**Table 3:** Parameters obtained by tensile testing of samples after 2 months.

	E (MPa)	Rm (MPa)	A (%)
A0 AKA0 AKA05	742.2 ± 43	16.6 ± 0.6	18.6 ± 0.7
	435.0 ± 20	10.4 ± 0.5	12.5 ± 4.7
	372.0 ± 25	9.1 ± 0.4	10.9 ± 2.6
AKA1 AKA2	367.5 ± 3.5	9.8 ± 0.7	12.0 ± 2.0
	364.7 ± 14	8.9 ± 1.2	9.2 ± 0.4

**Table 4:** Parameters obtained with nanoindentation of samples immediately after printing.

	E_R_ (GPa)	nH (MPa)
A0	4.4 ± 0.2	141 ± 14
AKA0	3.7 ± 0.2	109 ± 11
AKA05	5.1 ± 0.2	142 ± 10
AKA1	5.3 ± 0.4	159 ± 21
AKA2	3.6 ± 0.1	94 ± 8

## Data Availability

Data is contained within the article.
